# Computational identification of transcriptionally co-regulated genes, validation with the four ANT isoform genes

**DOI:** 10.1186/1471-2164-13-482

**Published:** 2012-09-15

**Authors:** Pierre-Yves Dupont, Audrey Guttin, Jean-Paul Issartel, Georges Stepien

**Affiliations:** 1 INRA, UMR 1019, Unité de Nutrition Humaine, 63122, St Genès-Champanelle, France; 2 Université d'Auvergne, Unité de Nutrition Humaine, Clermont Université, BP 10448, 63000, Clermont-Ferrand, France; 3 Institut des Neurosciences, Equipe Nanomédecine et Cerveau, Inserm U836, 38700, La Tronche, France; 4 Université Joseph Fourier 1, Grenoble, 38041, France; 5 Plate-forme Transcriptome et Protéome Cliniques, Institut de Biologie et Pathologie, CHU Grenoble, 38043, Grenoble, France; 6 CNRS, 38042, Grenoble, France

**Keywords:** Transcriptional regulation, Promoter analysis, Regulatory models, Adenine nucleotide translocator

## Abstract

**Background:**

The analysis of gene promoters is essential to understand the mechanisms of transcriptional regulation required under the effects of physiological processes, nutritional intake or pathologies. In higher eukaryotes, transcriptional regulation implies the recruitment of a set of regulatory proteins that bind on combinations of nucleotide motifs. We developed a computational analysis of promoter nucleotide sequences, to identify co-regulated genes by combining several programs that allowed us to build regulatory models and perform a crossed analysis on several databases. This strategy was tested on a set of four human genes encoding isoforms 1 to 4 of the mitochondrial ADP/ATP carrier ANT. Each isoform has a specific tissue expression profile linked to its role in cellular bioenergetics.

**Results:**

From their promoter sequence and from the phylogenetic evolution of these ANT genes in mammals, we constructed combinations of specific regulatory elements. These models were screened using the full human genome and databases of promoter sequences from human and several other mammalian species. For each of transcriptionally regulated *ANT1, 2 and 4* genes*,* a set of co-regulated genes was identified and their over-expression was verified in microarray databases.

**Conclusions:**

Most of the identified genes encode proteins with a cellular function and specificity in agreement with those of the corresponding ANT isoform. Our *in silico* study shows that the tissue specific gene expression is mainly driven by promoter regulatory sequences located up to about a thousand base pairs upstream the transcription start site. Moreover, this computational strategy on the study of regulatory pathways should provide, along with transcriptomics and metabolomics, data to construct cellular metabolic networks.

## Background

The metazoan genome is composed of coding sequences flanked by non-coding regions containing regulatory elements. These elements consist of short nucleotide sequences, which program gene expression at a given time and in a specific cell [1]. Despite the significant progress in genomics, which has led to the identification of most genes of the human genome, the knowledge of transcriptional regulation remains unclear. Our promoter study depends on a set of bioinformatics tools and allows the study of mechanisms of this genome regulation [2]. It relies on an analysis of gene promoter sequences which would include a combination (CRM, cis-regulatory modules) of several transcription factor binding sites (TFBS) ranging in length from ten to fifty nucleotides. TFBS are recognized by transcription factors (TFs), which allow the activation or repression of gene transcription. TFBS are difficult to differentiate from non-functional random genomic sequences [3]. These regulatory sequences and the genes that they govern define the functional spaces in the genome. Thus, such elements play a major role in the development, the environmental adaptation, the response to nutritional uptake and the pathogenesis.

The adenine nucleotide translocator (ANT), also referred to by the generic term "ADP/ATP carrier" (AAC), is a protein encoded from the nuclear genome and inserted in the inner mitochondrial membrane. ANT allows the exchange of ATP and ADP adenylic nucleotides between the mitochondrial matrix and the cytoplasm. Such a function is of primary importance because the ANT would be the main protein of the inner mitochondrial membrane able to convey this energy. The importance of this ANT protein is stressed by the fact that there exists, from yeasts to humans, four isoforms with similar amino-acid sequences (from 77 to 79% homology), with different kinetic properties, encoded from four independent genes, each with a specific expression depending on the nature of the tissue, cell type, developmental stage and status of cell proli-feration. This allows the energy production to adapt to the metabolic parameters linked to the cellular environment and cell cycle [4]. The peptide sequences of these four isoforms are very close (96% of homology); they differ only by several amino acids which are involved in the ATP and ADP interaction sites.

The specific transcriptional regulation of each of the four *ANT* genes is an interesting example of multi-isoform gene regulation. The metabolic and physiological consequences of these molecular regulatory mechanisms play a major role in the evolution of cellular metabolic pathways. Each of the four isoforms is known to play a specific role in cellular bioenergetics: ANT1 (SLC25A4) provides mitochondrial ATP for heart and skeletal muscle contraction [5]. The kinetic properties of this ANT1 isoform allow the rapid and massive mitochondrial ATP export required for muscular contraction. The second isoform, ANT2 (SLC25A5), is weakly or not at all expressed in human tissues and maintains the intra-mitochondrial functions under glycolytic conditions required in proliferative cells [6,7]. ANT2 is known to have a function opposite to that of ANT1 by transporting glycolytic ATP toward the mitochondrial matrix [5]. We identified a specific regulatory sequence in the promoter region of the human *ANT2* gene: the GRBOX element (Glycolysis Regulated Box) upstream of the TSS (transcription start site) [8]. ANT3 (SLC25A6) is the constitutively expressed ubiquitous isoform that is integrated into the mitochondrial membrane when no other isoform is produced [5]. In rodents, the *Ant3* gene was lost during evolution. It is possible that, unlike humans, rodent physiology does not require two isoforms with different kinetics (ANT1 and ANT3). This assumption would be supported by the disappearance, in the rodents, of the OXBOX regulatory element from the *ANT1* promoter [9], which would determine the muscle specific expression of this isoform. The last isoform, ANT4 (SLC25A31), was recently identified in humans, and is expressed mainly in the testicle [10]. This isoform appears in mammals and is essential during spermatogenesis [11]. Its peptide sequence is very similar (66-68% of identity) to that of the other ANT isoforms. The main characteristic of the ANT4 isoform is the presence of additional peptides, specifically the N- (13 amino acids) and C- (8 amino acids) terminal sequences, which the other three isoforms lack. The proposed hypothesis for the role of this isoform is that it compensates for the loss of the *ANT2* gene function (encoded by the X chromosome) during male meiosis [11]. A recent computational analysis enabled us to propose a specific role for the ANT4 isoform in spermatozoid bioenergetics [2].

We pursued and developed this computational analysis to compare the mechanisms of transcriptional regulation of the four ANT isoforms through analysis of nucleotide sequences upstream of the supposed TSS. The nucleotide sequences of these promoter regions from several mammalian species were compared to follow the phylogeny of specific sequences of transcriptional regulation. Promoter sequences preserved throughout evolution might be of major importance to the survival of the organism [12]. This study is based on a combination of software and databases including those available on-line, such as Genomatix [13] and EnsEMBL [14], GeneProm from our laboratory. This analysis led to interesting conclusions linking promoter structure and co-regulation of a set of genes.

## Results

**Figure 1:**
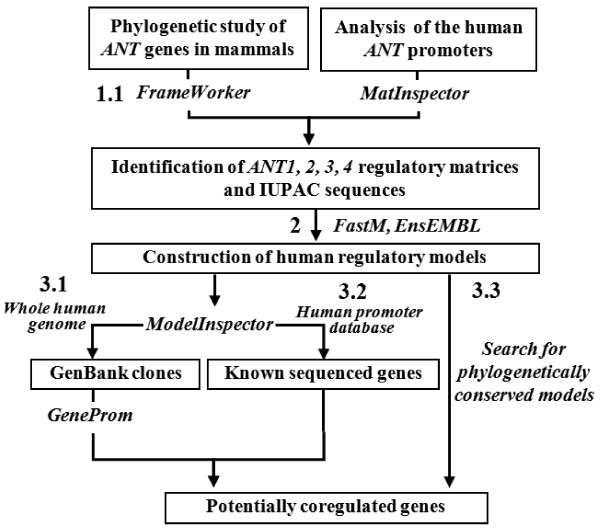
**Steps of the bioinformatics study.** 1.1 - Phylogenetic alignment of *ANT* genes in various mammalian species to filter unusable sequences and identification of matrices shared by aligned mammalian sequences from each ANT isoform gene (via Genomatix FrameWorker tool); 1.2 identification of TFBS in each human ANT isoform gene (MatInspector tool); 2 - Construction of regulation models by combining the selected matrices TFBS, IUPAC strings from bibliography; 3 - location of models in the whole human genome (3.1), in the ElDorado promoter database (3.2), and search for genes with models conserved in orthologous promoters (3.3); 4 - Identification of potentially co-regulated genes by GeneProm analysis.

An outline of the bioinformatics pipeline implemented for *ANT* sequences analysis is illustrated in Figure 1.

### Alignment and selection of ANT promoter sequences from various mammalian species

**Figure 2:**
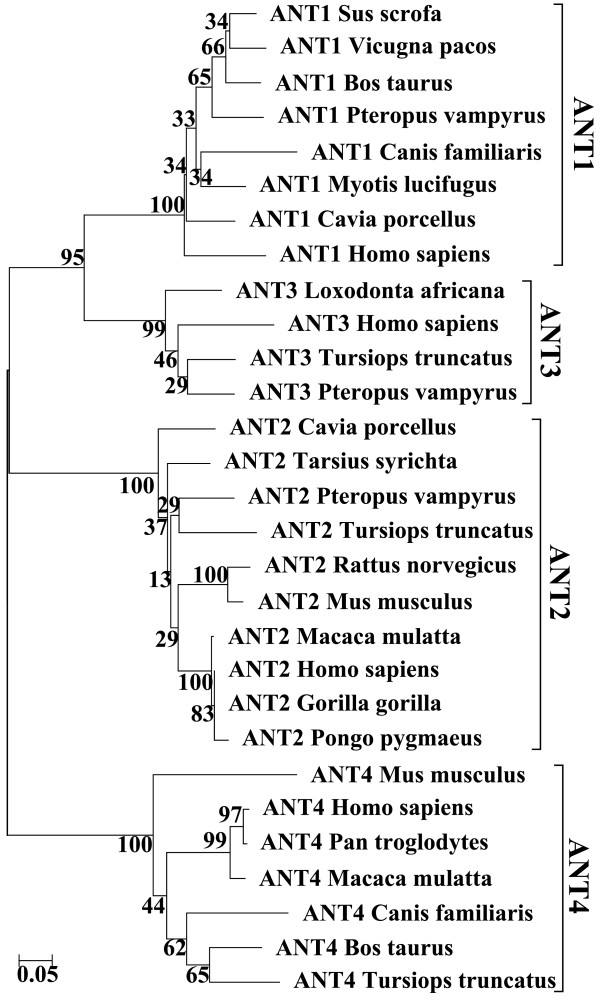
**Evolutionary relationships of taxa.** The evolutionary history was inferred using the Neighbor-Joining method [15]. The percentage of replicate trees in which the associated taxa clustered together in the bootstrap test (100 replicates) is shown next to the branches [16]. The tree is drawn to scale, with branch lengths in the same units as those of the evolutionary distances used to infer the phylogenetic tree. The evolutionary distances (number of base substitutions per site) were computed using the Maximum Composite Likelihood method [17]. The analysis involved 29 nucleotide sequences. All positions containing gaps and missing data were eliminated.

Mammalian species were screened for the homologous *ANT* gene sequences. Thirty mammalian species were screened for the four *ANT* genes and all *ANT* sequences (1500 nt upstream of the gene sequence and the first 500 nt of the gene including exon 1) were imported. The selected species corresponded to the 29 available mammalian sequences when the sequences were searched on EnsEMBL [14]. The mammalian sequences from each *ANT* gene were aligned using the CLUSTAL software and several sequences were selected according to the correct overlap of their 5’ upstream first exon sequences. Mammalian sequences with gaps, incomplete sequences or with more than 10% unknown nucleotides were discarded. Eight sequences (including Human) were obtained for the *ANT1* gene, 10 for the *ANT2* gene, 4 for the *ANT3* gene and 7 for the *ANT4* gene. The evolutionary history was inferred using the Neighbor-Joining method [15] with the bootstrap test [16] and with the Maximum Composite Likelihood method for evolutionary distances [17] (Figure 2; Additional file 1). Evolutionary analyses were conducted in MEGA5 [18].

### Identification of transcriptional matrices shared by the ANT1 to 4 gene sequences

**Figure 3:**
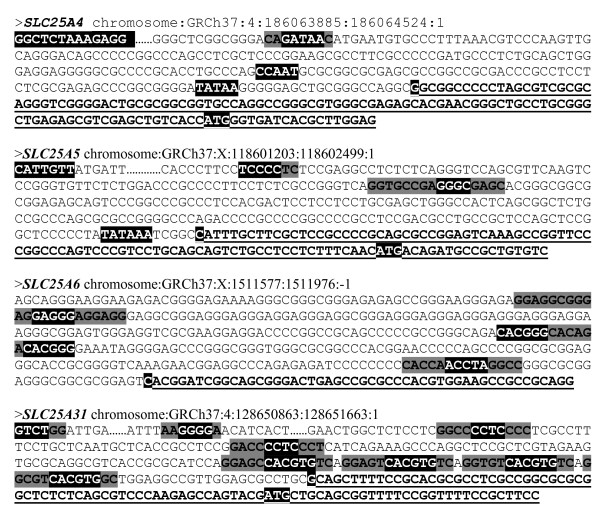
**Examples of human models constructed from the 4 *ANT *genes sequences.** Part of the promoter sequences and the beginning of the first exon (in bold and underlined) are shown for each isoform. Matrix core sequences, nucleotide strings and TSS are highlighted in black and matrix flanking sequences in grey. **A*NT1 *model**: GGCTCTAAA (OXBOX) / GATAA (V$GATA1.04) / CAAT / TATAA (O$VTATA.01) / G (TSS) /ATG; ***ANT2 *model**: CATTGTT (GRBOX) / TCCCC (V$MZF1.01) / GGGC (EGR1.02) / TATAA (O$VTATA.01) / G (TSS) / ATG; ***ANT3 *model**: GAGGG (V$CTCF) / CACGGG (V$CHRE) / ACCTA (V$RORA) / C (TSS); ***ANT4 *model**: GTCTGG (V$SMAD3.01) / GGGGA **(**V$MZF1.02) / CCCCTC (V$MAZ.01) / CACGTG (V$HIFF) / G (TSS)/ ATG.

Using the FrameWorker tool from Genomatix, we found several matrices shared by the selected mammalian promoter sequences of each *ANT* gene. Human models were constructed (Table 1) using different sets of parameters (number of matrices and nucleotide string copies, distance between matrices and nucleotide strings and stringency of each sequence). An example of model is shown in the promoter sequence of each isoform (Figure 3). The distance between the matrices and nucleotide strings was set to twice the distance given for the corresponding human *ANT* promoter by default.

**Table 1:** **Examples of * ANT *gene models in mammals**

	**Motif 1**	**Motif 2**	**Motif 3**	**Motif 4**	**Motif 5**
*ANT1*	OXBOX	V$GAT1. 04*	CAAT	O$VTATA.01	(TSS)
*ANT2*	GRBOX	V$MZF1.01	V$EGR1.02	V$SP1.01	O$VTATA.01
*ANT3*	V$CTCF*	V$CHRE*	V$RXRF*	V$RORA*	ATG
*ANT4*	V$SMAD3.01*	V$MZF1.02*	V$MAZ.01*	V$HIFF*	V$HIFF*

Matrix families (i.e. V$MEF2) or matrices (i.e. V$NRF2.01) with an asterisk were identified from phylogenetic analyses and showed to be conserved in orthologous promoters (Genomatix FrameWorker tool). The other matrices are selected with the Genomatix MatInspector tool on the human promoter sequences.

Most matrices and IUPAC strings from the *ANT1* models are directly or indirectly involved in muscle cell growth and differentiation. Most of those from the *ANT2* models found were involved in cell growth and proliferation ( Additional file 2). Based on phylogenetic analysis, transcriptional factors involved in *ANT3* gene regulation appear to serve different major functions. However, contrary to the three other *ANT* genes (1, 2 and 4), the stringency of the *ANT3* models required to identify co-regulated genes was too low to lead to conclusive results (i.e., the “Screening of co-regulated genes” part). Lastly, most matrices and IUPAC strings from the *ANT4* models are involved in testis development, spermatogenesis or the glycolytic energetic metabolism.

### Screening of genes co-regulated with each ANT

Constructed models of each *ANT* gene promoter were screened as described in Figure 1, either on the whole chromosome human sequences or the human promoter database (results with an asterisk) using Genomatix ModelInspector and GeneProm. Only genes with a model located next to their transcription start sites were selected (less than 500 bp of the 3’end of the model). A match for all matrices and IUPAC strings and in the same order is required. Moreover, several locations (one-third of the results) in clones without identified genes were not retained. Encoded protein data were obtained from the EnsEMBL database [14] and data on protein function were obtained from UniProt. [19] (UniProt ID: Q9H0H5).

Results with *ANT1* models (Table 2 and Additional files 3 and 4): twelve genes revealed from screening with the *ANT1* promoter models (ModelInspector / GeneProm analyses) were identified. All genes are specifically expressed in muscle or directly involved in bioenergetic metabolism. Seven of these genes were found to be overexpressed in microarrays data.

**Table 2:** **Genes co-regulated with* ANT1***

***Gene, ENSG ID***	**Encoded protein (EnsEMBL)**	**Protein function (UniProt)**
*ANO1*	Anoctamin-1	Ca^++^-activated chloride channel, higher levels in liver and **skeletal muscle**
*131620*		
*ARRDC3*	Arrestin domain-containing protein 3	Associated with plasma membrane, highly expressed in **skeletal muscle**
*113369*		
ATP5B*	ATP synthase, beta polypeptide	H + transporting, **mitochondrial** F1 complex
*110955*		
***ATP5D***	ATP synthase subunit δ, mitochondrial	**Mitochondrial** membrane ATP synthase
*99624*		
*ATP13A4*	Cation-transporting ATPase 13A4	ATP + H_2_O = ADP + phosphate; expressed in heart, placenta, liver, **skeletal muscles**
*127249*		
CBY1*	Protein chibby homolog 1	Expressed at higher levels in heart, **skeletal muscle**
*100211*		
COQ7*	Ubiquinone biosynthesis protein COQ7	Involved in ubiquinone biosynthesis, expressed in heart and **skeletal muscle**
*167186*		
COX6B2*	Cytochrome c oxidase subunit VIb 2	Connects the two **mitochondrial** COX monomers into the physiological form
*160471*		
COX7B*	cytochrome c oxidase subunit VIIb	One of the nuclear-coded polypeptide chains of **cytochrome c oxidase**
*131174*		
MYO1F*	Myosin IF	**Myosins** are actin-based motor molecules with ATPase activity
*142347*		
***NDUFA9***	NADH dehydrogenase 1 α- subcomplex, 9	Subunit of the **mitochondrial** membrane respiratory chain NADH dehydrogenase
*139180*		
NDUFS1*	NADH-ubiquinone oxido- reductase 75 kDa subunit	Subunit of the **mitochondrial** membrane respiratory chain NADH dehydrogenase
*23228*		

Several constructed models of the *ANT1* promoter region were screened as described in Figure 1 either on the full chromosomal human sequences or the human promoter library (results with an asterisk). Genes in bold are shown overexpressed in microarrays ( Additional file 3). Gene IDs are with 15 numbers (ex. ENSG00000023228).

Results with *ANT2* models (Table 3 and Additional files 3 and 4): seventeen genes were identified by ModelInspector / GeneProm analyses. Ten of them are expressed in conditions directly related to cell proliferation and glycolytic metabolism and five have a proposed function which appears to not be directly linked to one another. Twelve genes were found to be overexpressed in microarrays data (over the fifteen with a correct probe set on Affymetrix chips).

**Table 3:** **Genes co-regulated with* ANT2***

***Gene, ENSG ID***	**Encoded protein (EnsEMBL)**	**Protein function (UniProt)**
*AURKC*	Serine/threonine-protein kinase 13	Organizing microtubules during **mitosis** and chromosome segregation
*105146*		
***BTG1***	B-cell translocation gene 1 protein	Anti-proliferative protein, associated with the **G1 phase** of the cell cycle
*133639*		
*CDKN2AIP*	CDKN2A-interacting protein	Activates **p53**/TP53 by CDKN2A-dependent and independent pathways
*168564*		
***CEBPB***	CCAAT/enhancer-binding protein beta	Transcriptional activator of genes involved in immune responses
*172216*		
*CKB*^δ^	Creatine kinase B-type	**Transfer of phosphate** between ATP and various phosphogens
*166165*		
***COL1A1***	Collagen alpha-1(I) chain	Type I collagen is a member of group I collagen
*108821*		
DDIT4L*	DNA damage-indu- cible transcript 4	**Inhibits cell growth** by regulating the TOR signaling pathway
*145358*		
***DKK1***	Dickkopf-related protein 1	Antagonizes Wnt signalling by inhibiting LRP5/6 interaction with Wnt
*107984*		
FGL1*^δ^	Fibrinogen-like protein 1	Hepatocyte **mitogenic** activity
*104760*		
***GADD45B***	Growth arrest inducible GADD45 β	**Regulation of growth** - apoptosis, activation of stress-responsive genes
*99860*		
***GDF15***	Growth/differentiation factor 15	Transforming **growth factor** beta receptor signalling pathway
*130513*		
***HIF1A***	Hypoxia-inducible factor 1-alpha	Master transcriptional regulator of the **adaptive response to hypoxia**
*100644*		
***KCNJ8***	ATP-sensitive rectifier K^+^ channel 8	This potassium channel is controlled by G proteins
*121361*		
LY96*	Lymphocyte antigen 96	Cooperates with TLR4 in response to bacterial lipopolysaccharide
154589		
*NPPC*	C-type natriuretic peptide	**Proliferation**/differentiation regulation of growth plate chondrocytes
*163273*		
***RARRES1***	RAR responder protein 1	Negative regulation of **cell proliferation**
*118849*		
***SMCHD1***	Chromosome domain maintenance	**ATP binding**
*101596*		

Several constructed models of the *ANT2* promoter region were screened as described in Figure 1 either on the whole chromosome human sequences or the human promoter library (results with an asterisk). Function involved in cell proliferation is shown in bold characters. Genes in bold are shown overexpressed in microarrays ( Additional file 3). ^δ^No correct probe set on Affymetrix chips corresponding to this gene. Gene IDs are with 15 numbers (ex. ENSG00000023228).

Results with *ANT3* models (Additional files 4 and 5): no candidate genes were identified with regular stringency (set as the same level of that used for the three other *ANT* sequences). A lower stringency led to identify 10 genes with the ModelInspector and GeneProm analyses. Five have unknown function and the remaining 5 have heterogeneous functions not linked to bioenergetic pathways. The sole gene found overexpressed in testes microarrays, IFT88, encodes for an intraflagellar transport protein involved in cilium biogenesis. This gene appears as a false positive result.

Results with *ANT4* models (Table 4 and Additional files 3 and 4): 21 genes were identified by ModelInspector/GeneProm analyses. Four of these genes have an unknown or a supposed function; 17 are specifically expressed in the testis, during spermatogenesis, or involved in prostate metabolism. Thirteen genes were found to be overexpressed in microarrays data (over the twenty with a correct probe set on Affymetrix chips).

**Table 4:** **Genes co-regulated with* ANT4***

***Gene, ENSG ID***	**Encoded protein (EnsEMBL)**	**Protein function (UniProt)**
***AMDHD2***	Acetylglucosamine-6-phosphate deacetylase	N-acetyl-D-glucosamine 6-P + H_2_O = D-glucosamine 6-P + acetate
*162066*		
APEX1*	APEX nuclease (DNA repair enzyme) 1	Repair of apurinic / apyrimidinic sites **in testis**
*100823*^*§*^		
CDK4*	cyclin-dependent kinase 4	Cell cycle G1 phase progression **in male reproduction**
*135446*^*§*^		
CLPB*	Caseinolytic peptidase B protein homolog	Function as a **regulatory ATPase** and related to protein secretion
*162129*		
***FIP1L1***	Pre-mRNA 3'-end-processing factor FIP1	Contributes to poly(A) site recognition and poly(A) addition
*145216*		
*FLJ32713*^*δ*^	Unknown **(TESTI2000756)**	Unknown (**expressed in testis**)
*fis*^***§***^		
FNDC3A*	Fibronectin type-III domain-containing 3A	Mediates spermatid-Sertoli adhesion during **spermatogenesis**
*102531*		
G6PC2*	Glucose-6-phosphatase 2	Glucose production, expressed **in testis**
*152254*		
G6PC3*	Glucose-6-phosphatase 3	Glucose production in endoplasmic reticulum, expressed **in testis**
*141349*		
KAT5*	K(lysine) acetyltransferase 5	Chromatin remodelling with an abundant **spermatid** protein
*172977*^*§*^		
KLHL12*	Kelch-like protein 12	Ubiquitin-protein E3 ligase complex adapter, highly expressed **in testis**
*117153*		
LAMP1*	lysosomal-associated membrane protein 1	Binds amelogenin, differentially expressed in **spermiogenesis**
*185896*^*§*^		
RPUSD4*	RNA pseudouridylate synthase domain-containing	Unknown, **expressed in prostate**
*165526*^*§*^		
*SLC2A4*	solute carrier family 2, member 4 (GLUT4)	Facilitated glucose transporter, **detected in human testis**
*181856*^*§*^		
***SOHLH1***	**spermatogenesis** and oogenesis specific HLH1	Germ cell-specific, oogenesis regulator and **male germ cells**
*165643*^*§*^		
*SUN1*	chr. 7 unc-84 homolog A	Nuclear anchorage/migration, expres- sion of **meiotic reproductive genes**
*164828*^*§*^		
TDRD1*	tudor domain containing 1	Essential for **spermiogenesis**
*95627*^*§*^		
THAP8*	O-sialoglycoprotein endopeptidase	Unknown (**TESTI2004929)**
*161277*^*§*^		
***TKTL1***	transketolase-like 1	Important role in transketolase activity, **testis** expressed
*7350*^*§*^		
*TMEM184A*	transmembrane protein 184A = Sdmg1	**Male-specific** expression in embryonic gonads
*164855*^*§*^		
UBE2B*	ubiquitin-conjugating enzyme E2B	Post-replicative DNA damage repair **in spermatogenesis**
*119048*^*§*^		

Several constructed models of the *ANT4* promoter region were screened as described in Figure 1 either on the full chromosomal human sequences or the human promoter library (results with an asterisk). Function involved in spermatogenesis or in testis or prostate metabolism is shown in bold characters. Genes in bold are shown overexpressed in microarrays ( Additional file 3). ^***§***^Genes identified in the previous ModelInspector / GeneProm analysis [2]. ^δ^No correct probe set on Affymetrix chips corresponding to this gene.

If numerical expression results were compared for the 4 ANT isoform genes (scatter plots in Additional file 4), the expression levels were similar in glioblastoma versus control tissue for the ubiquitous ANT3 isoform gene and the identified co-regulated genes. In the opposite, the regulated *ANT1, 2* and *3* genes were over-expressed in the corresponding tissues (muscle for ANT1, glioblastoma for ANT2 and testis for ANT4)

## Discussion

We built a pipeline of bioinformatics analyses for the study of the transcriptional regulation of a set of genes and the prediction of co-regulated genes. Co-regulated genes could encode proteins involved in the same metabolic network including an entire set of different pathways. This analysis was designed for, in the longer term, predicting a precise signature of a cellular metabolic change, which is the consequence of physiological conditions, disease, or a response to a specific pharmacological or nutritional treatment. This pipeline can analyse the structure of the promoter region of a gene and the construction of regulatory models composed of a combination of several small nucleotide sequences specifically linked to the gene function. The strategy of the promotology analysis that we adopted relies on the crossing of three complementary analyses: 1 – screening of genes located next to the constructed models on the whole human genome: combination of the Genomatix ModelInspector tools and GeneProm software) [2]; 2 – screening of these models on a database of human promoter sequences by ModelInspector / Human Promoters; 3 - screening of selected models in several mammalian species using the “Search for phylogenetically conserved promoter models” tool). The crossing of these three analyses allowed us to identify, with higher stringency, a limited set of genes controlled by the same model of promoter sequence.

**Figure 4:**
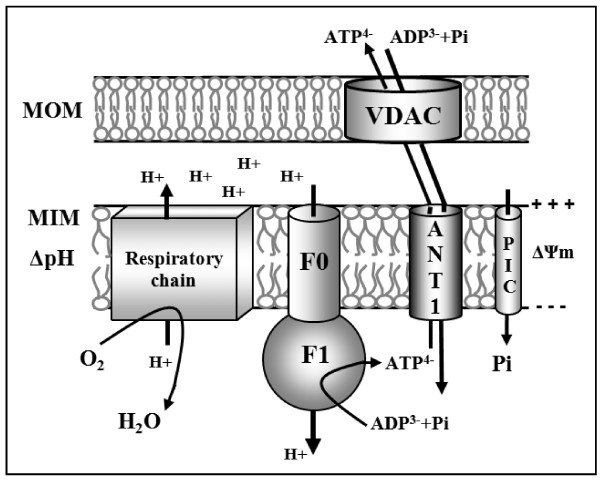
**Schematic representation of the role of ANT1 isoform in muscle cell oxidative phosphorylation.** ADP^3-^ and inorganic phosphate (Pi) are transported across the mitochondrial inner membrane (MIM) into the mitochondrial matrix by the mitochondrial ANT and phosphate carrier (PiC), respectively. F1F0-ATPase combines Pi and ADP to form ATP, which is then exchanged for ADP across the MIM by ANT1 then across MOM (mitochondrial outer membrane). The whole reaction is driven by a proton gradient maintained mainly by the respiratory chain. Six of the genes identified from our promotology analysis encode proteins included in the oxidative phosphorylation (respiratory chain and F0-F1 ATP synthase proteins).

This bioinformatics protocol was tested on a set of genes encoding four isoforms of the ANT protein (adenine nucleotide translocator), each having a specific role in a specific cellular type. Three of these four proteins (ANT1, ANT2 and ANT4) are controlled at the transcriptional level by a specific mechanism. The fourth (ANT3) is the ubiquitous isoform constitutively expressed in all cells. The implementation of our promotology analysis on this set of four *ANT* genes accounted for a powerful validation of our strategy:

*ANT1:* the gene encoding the isoform specifically expressed in muscle tissues [5] enabled us to build five models of the promoter sequence (Additional file 2). These models are found in the promoters of 12 genes highly expressed in muscle tissues and / or involved in bioenergetic metabolism and having a direct connection with muscle cell metabolism or mitochondrial ATP synthesis (Table 2). In particular, 6 of these genes encode proteins that are included to 3 of the mitochondrial complexes of oxidative phosphorylation: NADH dehydrogenase (complex I), cytochrome oxidase (complex IV) and ATP synthase (complex V) [20] (Figure 4). Moreover, another gene carrying a model, COQ7, is involved in the synthesis of these complexes [21]. Other identified genes encode proteins involved in major pathways of muscle metabolism such as ANO1/TMEM16A in calcium transport [22] and MYO1F in muscle contraction [23].

**Figure 5:**
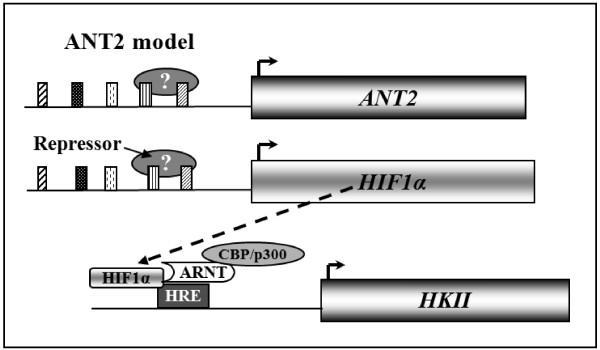
**Schematic representation of the proposed indirect *HKII */ *ANT2 *co-regulation in glycolytic conditions.** In glycolytic conditions, the mitochondrial hexokinase isoform, HK II, generates ATP from cytoplasmic glucose 6-P (G-6P). The ATP^4−^ is then imported into mitochondria by the ANT2 isoform, contributing to the maintenance of the mitochondrial membrane potential (ΔΨm). The HKII gene transcription is induced by the HIF1-alpha protein. CBP/p300, CREB-binding protein; ARNT, aryl hydrocarbon nuclear translocator; HRE, hypoxia response element.

*ANT2:* Most of the 17 genes carrying a model resulting from the *ANT2* gene encode proteins that play a role in pathways related to cell division and proliferation (AURKC, BTG1, FGL1, GDF15, NPPC) (Table 3). Several other identified genes encode signalling proteins such as CDKNÀIP, GADD45B or HIF1-alpha. The HIF1-alpha-protein is known to induce the transcription of the HKII gene under conditions of glycolytic metabolism [24,25]. Similar to ANT2, HKII is involved in the uptake of glycolytic ATP through the inner mitochondrial membrane. This *HKII* / *ANT2* indirect co-regulation is consistent with their complementary roles in glycolytic conditions (Figure 5) [4,7,8].

*ANT3:* No gene carrying models built from the *ANT3* gene promoter could be identified if stringency parameters similar to that used for three other isoforms were selected. Analyses with lower stringencies (lower number of matrices in models and lower similarity of matrices or IUPAC strings compared with the three other *ANT* genes) reveal up to 10 genes. Five of these genes encode proteins with an unknown function; the five others have no apparent functional link among one another (Additional file 5). Moreover, the analysis of the *ANT3* promoter region by the Genomatix MatInspector and PromoterInspector tools proposed few regulation matrices as compared to genes of the three other regulated isoforms (result not shown). In low stringency conditions, the sequence of the model becomes not significant: for a model with 5 matrices, the 4 to 6 nucleotides of the core sequences are conserved with an identity of 100%. Thus, such models could often lead to the identification of unrelated false positive genes. Moreover, this study confirms that our model construction from conserved models in different mammalian species could clearly identify the not specifically regulated ubiquitous genes.

**Figure 6:**
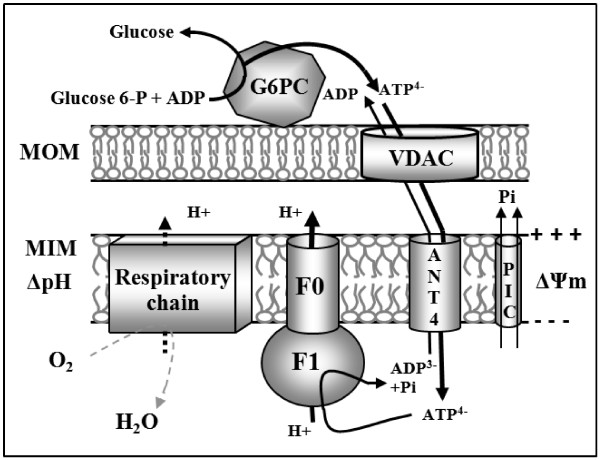
**Schematic representation of the role of ANT4 isoform in bioenergetics during spermatogenesis.** The glucose-6-phosphatases 2 and 3 (G6PC) generates ATP from glucose 6-P (G-6P) produced by the cytoplasmic hexokinase, HK. The ATP^4−^ is imported into mitochondria across the MOM (mitochondrial outer membrane) through the voltage-dependent anion channel (VDAC), and then across the MIM (the mitochondrial inner membrane) by the ANT4 isoform. ATP^4−^ contributes to the maintenance of the mitochondrial membrane potential (ΔΨm) in spermatozoid mitochondria. The hydrolysis of imported glycolytic ATP^4−^ by the F1 component of the ATP synthase leads to 1 - the release of ADP^3−^ in mitochondria with the gain of a negative charge on the matrix side; and 2 - the ejection of a proton into the intermembrane space through the F0 component.

*ANT4:* The models constructed from the *ANT4* gene promoter allowed us to identify 15 genes with known function in spermatogenesis (Table 4). Our previous work on the promoter of this *ANT4* gene led us to identify a part of these co-regulated genes [2]. The new version of our GeneProm software enabled us to identify 5 new genes also directly related to spermatogenesis. Among these 5 genes specifically expressed in the testes, two encode the glucose-6-phosphatases 2 and 3 enzymes, producing glucose from glucose-6-phosphate, leading to ATP synthesis [26]. This function is in complete agreement with the exclusively glycolytic metabolism of spermatozoids [11]. Moreover, this glycolytic ATP production is also consistent with the expression and the specific role of the ANT4 isoform in spermatozoid bioenergetics: part of the ATP produced by glucose-6-phosphatases 2 and 3 could be imported into mitochondria by ANT4 (gene located on chromosome 4) to compensate for the absence of the ANT2 isoform (gene located on chromosome X and not transcribed during spermatogenesis) (Figure 6) [2].

Thus, this *in silico* analysis leads to very interesting conclusions on the relationship between transcriptional regulatory pathways and protein function in a cellular metabolic network. A set of genes encoding protein isoforms expressed with tissue specificity turned out to be a clear validation of our strategy. The phylogenetic comparison of promoters allowed for the identification of nucleotidic matrices with major roles in gene regulation. However, the exclusive use of regulatory matrices has limits; it requires multiple analyses with different parameters of stringency and layout of each matrix compared with the other involved regulatory elements. The presence, in a promoter region, of described and validated nucleotide sequences, such as the OXBOX (oxidative box) [9] and GRBOX (glycolysis regulated box) [8], known to take part to the regulation of the *ANT1* and *ANT2* genes, respectively, is a crucial argument in the construction of a powerful regulatory model. The presence of a gene encoding a ubiquitous isoform, ANT3, and the failure to identify co-regulated genes with models built from its promoter region allow us to validate our strategy. Moreover, this analysis of an unregulated ubiquitous gene becomes a standard for further works by providing a precise scale of similarity of matrices in our models. Thus, a strategy of promotology, based simultaneously on a conclusive phylogenetic analysis and on already validated regulatory nucleotide sequences, allows for the identification of co-regulated genes.

Our work on this set of isoforms also showed that transcriptional regulation is a major mechanism of cellular specificity. The structure of the promoter sequence directly upstream of the transcription start site itself allows the identification of co-regulated genes. This suggests that, at least for the regulation of bioenergetic pathways described in this work, other supposed regulatory mechanisms including microRNA or the messenger RNA stability, would intervene in other cellular functions. Moreover, our strategy allows overcoming the insufficiencies of other techniques used for the study of gene expression. Taking into account the very close coding sequences of the four ANT isoforms, no currently commercial microarrays able to simultaneously and specifically quantify the four transcripts is available. In addition, the very hydrophobic properties of these proteins do not allow their identification by 2D electrophoresis and no specific antibody of each isoform is available.

## Conclusion

In conclusion, our computational strategy on a set of four isoforms, known for their specific functions in cell bioenergetics, enabled us to develop a powerful analysis of gene promoter sequences. Our analyses enabled us to identify an entire set of co-regulated genes, involved in the same cellular function. This study validates the major role of the proximal promoter region in tissue specificity and should provide, along with transcriptomics and metabolomics, assistance in developing cellular metabolic networks and the study of their regulatory pathways.

## Methods

### Process of bioinformatics study

Our study was performed in four steps (Figure 1): 1 - We began with a short phylogenetic study of the four *ANT* genes from various mammalian species. This step enabled us to check database annotations and eliminate sequences that were impossible to align to the four human *ANT* genes. The application of the Genomatix tool FrameWorker to the selected mammalian sequences provided a list of regulatory elements (matrices) or matrix families identified among the aligned sequences and conserved in orthologous promoters. The Genomatix MatInspector tool proposes a set of matrices along the promoter sequence of each human *ANT* gene. 2 - We organized matrices, already known TFBS and canonical sequences (CAAT, TATAA, ATG) into regulatory models by the Genomatix FastM tool. 3 - Models were then screened with the Genomatix ModelInspector tool across the entire human genome (GenBank clones [27]) and in the human promoter database Genomatix Eldorado. Models with positive results were screened in 12 mammalian promoter databases by the Genomatix “Search for phylogenetically conserved models” tool. 4 - Genes were identified from their position in GenBank clones using the GeneProm software we deve-loped [2] and allowing to identify all human genes (known and supposed) with a model located upstream. The GeneProm software consists of a web application accessible on SourceForge and is distributed under MIT license. It was developed in Ruby by using the Rails framework and in Perl by using the Bioperl and EnsEMBL libraries.

### Phylogenetic study of the ANT genes in mammals

Nucleotide sequences of the four *ANT* genes were screened in 29 mammalian species including humans. Sequences were imported from the EnsEMBL database [14]. These sequences included the first 500 bases of the gene, including exon 1, and an additional 1500-nt sequence upstream of the TSS. Manual sorting of these sequences was performed by withdrawing the sequences containing either a high number of undefined bases (more than 10% within the promoter region) or annotation errors at the transcription initiation sites (identified by alignment of the sequences, including the first exon for each species). A phylogeny reconstruction of the retained coding sequences was performed to validate the functional annotations of these genes. The alignment algorithm used was CLUSTAL 2.0, using the default settings. The phylogeny was rebuilt using two different methods: the neighbour-joining method with minimum evolution and UPGMA (Unweighted Pair Group Method with Arithmetic mean) both with bootstrap evaluation. Both techniques provided concordant results. This phylogeny was carried out to verify the annotations of the sequences in the databases and the promoter sequences to be compared.

After considering the phylogenetic results of our study, we decided to analyze only a small number (29) of mammalian sequences that did not contain undetermined bases in their promoter regions and did not significantly diverge from the human sequence (Figure 2 and Additional file 1). Additionally, the transcription initiation sites of all the retained sequences were compatible with the known human initiation site. The alignment of the 5' sequences (1500 nt upstream of the ATG site) was performed using the CLUSTAL 2.0 software. Similar alignments have been obtained using MUSCLE. The sequences of the four *ANT* genes (*ANT1 to 4* aliases *SLC25A4, SLC25A5, SLC25A6, SLC25A31, respectively*) were aligned to verify their annotation in the EnsEMBL database [14]. Thus, the list of 15 selected species takes into account the sequencing quality of this genomic region.

### Identification of transcriptional matrices

The FrameWorker tool of the Genomatix software package was used for this part of the analysis. This tool identified transcriptional regulatory sequences from the bibliography, called regulatory matrices, which were shared by a set of gene promoter sequences. Matrices were identified from the Genomatix database produced by the alignment of all regulatory elements identified to date in all vertebrates. The database includes 727 vertebrate matrices classified into 170 families (noted V$) and 16 additional general matrices from higher organisms, classified into ten families (noted O$). Additionally, the FrameWorker tool enables identification of the regulatory models shared by different promoter sequences. The MatInspector tool simultaneously allows the identification of the regulatory matrices within a promoter sequence and the analysis of different parameters.

### Construction of regulatory models and screening

The nucleotide distances between the different identified matrices of the most relevant models were then bound between minimal and maximal values. Previously identified and potentially interesting nucleotide sequences in the promoter area of interest were then combined with the set of selected matrices. The resulting new model, which generally included 4 to 6 matrices or nucleotide strings (Table 1), could be searched using the selected stringencies in three databases of mammalian genomes.

1 - The whole human genome (GenBank Release 184) using the Genomatix ModelInspector tool [28]. A list of clones (contigs) containing the studied model was obtained in this analysis with the exact positions of the identified models and their orientation in each clone; 2 - The Genomatix Eldorado 08–2011 database of human promoter sequences including a set of approximately 120.000 promoter sequences associated with transcripts; 3 - The Genomatix Eldorado 08–2011 database of a set of mammalian promoter sequences. The “Search for phylogenetically conserved promoter models” tool allows a search for models that are conserved in orthologous promoter sequences of several mammalian species, which provides evidence for the functionality of promoter models through their preservation during evolution.

The different stringency parameters of this study (ModelInspector parameters) were as follows: maximum number of mismatches allowed for either matrix or nucleotide string; threshold (number of matrices or strings present in the sequence *vs.* the number of matrices or strings to find); research of individual or matrix families; global and core matrix similarities; matrix sense in relation to the clone; and minimal and maximal distances between two matrices or nucleotide strings.

### Identification and selection of genes

**Figure 7:**
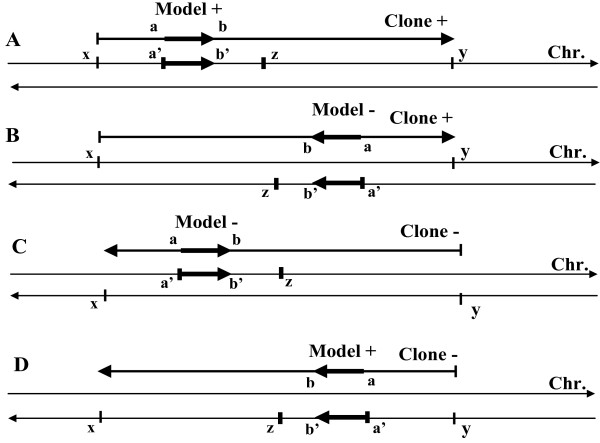
**GeneProm filters for the respective orientations of the model, the identified gene, the EMBL contig and the chromosomal DNA strand.** 2000 nt was the maximal distance between the 5’ end of the model and the TSS of a gene. A (model + / clone + / chromosome +): a’ = x + a and z = x + a + 2000; B (−/+/−): a’ = x + a and z = x + a – 2000; C (− /- / +): a’ = y - a and z = y – a + 2000; D (+/−/-): a’ = y - a and z = y – a – 2000.

The list of GenBank clones proposed by ModelInspector was exported into our GeneProm software. GeneProm allowed us to find the model chromosomal location and to identify known, supposed or unknown genes present in the immediate proximity (or in partial overlap) of the considered model (Figure 7). The filters used in this analysis were: 1 - the respective orientations of the model, the EMBL contig, the chromosomal DNA strand and the identified gene; 2 – the maximal distance from the 3’ end of the model to the TSS of the gene (500 nt by default); 3 - the maximal length of the overlapped sequence between the model and the 5’ end sequence of the identified gene (200 nt by default). These filters permitted the selection of genes directly downstream of the researched model from the same strand of DNA with the same orientation.

The list of the genes obtained from the two analyses (ModelInspector on GenBank and on Eldorado database) was manually analysed, and an exhaustive bibliography was obtained for each gene (HGNC database [29]) to identify the function of each corresponding protein and their direct or indirect links with the ANT protein. The GeneProm software was then paired using a script to the KEGG database [30], allowing the identification of the metabolic networks involved in the protein encoded from each gene identified from the previous step. A complementary bibliographic analysis was performed to relate the identified proteins to suggested metabolic pathways. Key proteins involved in these pathways could be identified.

### Gene expression from Affymetrix Human Genechips

Gene expression data were downloaded from the GEO database [31]. Data from glioblastoma tissues, normal skin, normal muscle, normal testis and normal brain tissues came from the series GSE15824, GSE7307 and GSE14905 and were all measured by hybridization with Affymetrix Human Genechips U133 plus 2.0. Data from all the different samples were normalized by quantile method with “Affy package on bioconductor”. The ability of all the probe sets of the Affymetrix Genechips to hybridize to their target mRNAs was ascertain using the ADAPT database [32]. Values reported for all the individual tissues correspond to the averaged values of the expression levels of the probe set in all the samples of a given tissue. Each probe set underwent statistical analysis using the Wilcoxon Test.

## Abbreviations

ANT: adenine nucleotide translocator; CRM: cis-regulatory modules; nt: nucleotides; TFBS: transcription factor binding sites; TFs: transcription factors; TSS: transcription start site.

## Competing interests

The authors declare that they have no competing interests.

## Authors’ contributions

P-YD carried out the GeneProm software construction, the phylogenetic analysis and helped to draft the manuscript. AG carried out the analysis of the Affymetrix Human Genechips and the scatter plots of the expression levels, J-PI participated to the design of the study and to the Genechips selection, GS conceived the study, carried out the computational study, followed its design and coordination, and drafted the manuscript. All authors read, corrected and approved the final manuscript.

## Supplementary Material

Additional file 1 ***ANT *gene sequences in mammals.** Mammalian *ANT* gene sequences selected for the 4 ANT isoforms in 24 mammals. Sequences were extracted from EnsEMBL database. Sequences in bold are sequences that do not contain undetermined bases in their promoter and are not too divergent from the corresponding human sequence. Click here for file

Additional file 2 ***ANT *gene models in mammals.** Matrix families (i.e. V$MEF2), matrices (i.e. V$NRF2.01) and IUPAC strings from the four human ANTs gene promoters. For matrix families, an example of IUPAC string is shown. For matrices, only nucleotides (IUPAC) with high information content are presented (the matrix exhibits a high conservation at this position). For matrices, nucleotides in bold capital letters denote the core sequence used by MatInspector (defined as the highest, usually four, conserved consecutive positions) [33]. The other nucleotides are from the flanking sequence. Motifs with an asterisk were identified from phylogenetic analyses. IUPAC nucleotide code: R (A or G); Y (C or T); S (G or C); W (A or T); K (G or T); M (A or C); B (C or G or T); D (A or G or T); H (A or C or T); V (A or C or G); N (any base). Click here for file

Additional file 3 **Scatter plots of the expression levels of genes with identical promoter models with the *ANT *promoters in different tissues.ANT1:** average expression levels of the genes from Table 2 in the lung, skin and brain tissues versus their expression in muscle. **ANT2:** average expression levels of the genes from Table 3 in normal brain tissue versus their expression in glioblastoma. **ANT3:** average expression levels of the genes from Additional file 5 in normal brain tissue versus their expression in glioblastoma. **ANT4:** average expression levels of the genes from Table 4 in the lung, skin and brain tissues versus their expression in testis. Red diamonds correspond to over-expressed genes with at least a 1.5 fold changes between tissues. Click here for file

Additional file 4 **Genes co-regulated with the ANT3 gene.** The full set of results obtained from the analysis with all constructed models of the *ANT3* promoter regions were screened as described in Figure 1 either on the whole chromosome human sequences or the human promoter library (results with an asterisk). The gene in bold is shown overexpressed in microarrays. Gene IDs are with 15 numbers (ex. ENSG00000023228). Click here for file

Additional file 5 **Expression levels of genes that share promoter models with *ANT *genes in different tissues.** Expression levels of genes that share promoter models with ANT promoters in different tissues. Data were obtained from hybridization experiments using Affymetrix genechips as described in material and methods. Y scale in log2 of the average values of the ratios. An average value of the ratios was also calculated for each gene whose expression was assessed by several probe sets. Click here for file
