# Supplementary material for: Computational identification of transcriptionally co-regulated genes, validation with the four ANT isoform genes
Source: BMC Genomics. 2012 Sep 15;13:482. doi: 10.1186/1471-2164-13-482 (PMC3477019; doi:10.1186/1471-2164-13-482)
Supplement: Additional file 1 — ANT gene sequences in mammals. Mammalian ANT gene sequences selected for the 4 ANT isoforms in 24 mammals. Sequences were extracted from EnsEMBL database. Sequences in bold are sequences that do not contain undetermined bases in their promoter and are not too divergent from the corresponding human sequence. [file 1471-2164-13-482-S1.docx]

**Additional file 1. *ANT* gene sequences in mammals**

| ***Species* (common name)** | **Sequences of ANT genes** |
| --- | --- |
| *Homo sapiens* (Human) | ***ANT1 (ENSG00000151729); ANT2 (ENSG00000005022 ;***  ***ANT3 (ENSG00000169100); ANT4 (ENSG00000151475)*** |
| *Bos Taurus* (ox) | ***ANT1 (ENSBTAT00000017580);*** *ANT2 (ENSBTAG00000046037)****;***  ***ANT4 (ENSBTAG00000012826)*** |
| *Canis familiaris* (dog) | ***ANT1 (ENSCAFG00000007596);*** *ANT2 (ENSCAFG00000018384)****;***  *ANT3 (ENSCAFG00000010987)****; ANT4 (ENSCAFG00000003924)*** |
| *Cavia porcellus* (guinea pig) | *ANT1 (ENSCPOG00000005275); ANT2 (ENSCPOG00000009202)* |
| *Echinops telfairi* (hedgehog) | *ANT1 (ENSEEUG00000001021)* |
| *Felis catus* (cat) | *ANT1 (ENSFCAG00000007057); ANT2 (ENSFCAG00000005481);**ANT3 (ENSFCAG00000001211)* |
| *Gorilla gorilla* (gorilla) | *ANT2 (ENSGGOG00000014279)* |
| *Loxodonta africana* (elephant) | *ANT3 (ENSLAFG00000001584)* |
| *Macaca mulatta* (macaque) | ***ANT2 (ENSMMUG00000022663);*** *ANT3 (ENSMMUG00000006899);*  ***ANT4 (ENSMMUG00000015243)*** |
| *Mus musculus* (mouse) | ***ANT2 (ENSMUSG00000016319); ANT4 (ENSMUSG00000069041)*** |
| *Myotis lucifugus* (microbat) | *ANT1 (ENSMLUG00000003712)* |
| *Nomascus leucogenys* (gibon) | *ANT1 (ENSNLEG00000011142)* |
| *Otolemur garnettii* (galago) | *ANT4 (ENSOGAG00000005752)* |
| *Pan troglodytes*(chimpanzee) | *ANT3 (ENSPTRG00000029304);* ***ANT4 (ENSPTRG00000016432)*** |
| *Pongo pygmaeus* (orangutan) | *ANT2 (ENSPPYG00000020669); ANT3 (ENSPPYG00000019151)* |
| *Procavia capensis* (hyrax) | *ANT3 (ENSPCAG00000014264)* |
| *Pteropus vampyrus* (megabat) | *ANT1 (ENSPVAG00000015279); ANT2 (ENSPVAG00000014924);* *ANT3 (ENSPVAG00000004356); ANT4 (ENSPVAG00000002926)* |
| *Rattus Norvegicus* (rat) | *ANT2 (ENSRNOG00000039980)* |
| *Sus scrofa* (pig) | *ANT1 (ENSSSCG00000015790)* |
| *Tarsius syrichta* (tarsier) | *ANT1 (ENSTSYG00000004233; ANT2 (ENSTSYG00000002246)* |
| *Tupaia belangeri* (tree shrew) | *ANT1 (ENSTBEG00000011502)* |
| *Tursiops truncatus* (dolphin) | ***ANT2 (ENSTTRG00000001007); ANT3 (ENSTTRG00000015399);***  ***ANT4 (ENSTTRG00000012978)*** |
| *Vicugna paosc* (alpaca) | *ANT1 (ENSVPAG00000008737); ANT2 (ENSVPAG00000000644)* |

Mammalian *ANT* gene sequences selected for the 4 ANT isoforms in 23 mammals including Human. Sequences were extracted from EnsEMBL database. Sequences in bold are sequences that do not contain undetermined bases in their promoter and are not too divergent from their corresponding human sequence.
