# Supplementary material for: Computational identification of transcriptionally co-regulated genes, validation with the four ANT isoform genes
Source: BMC Genomics. 2012 Sep 15;13:482. doi: 10.1186/1471-2164-13-482 (PMC3477019; doi:10.1186/1471-2164-13-482)
Supplement: Additional file 2 — ANT gene models in mammals. Matrix families (i.e. V$MEF2), matrices (i.e. V$NRF2.01) and IUPAC strings from the four human ANTs gene promoters. For matrix families, an example of IUPAC string is shown. For matrices, only nucleotides (IUPAC) with high information content are presented (the matrix exhibits a high conservation at this position). For matrices, nucleotides in bold capital letters denote the core sequence used by MatInspector (defined as the highest, usually four, conserved consecutive positions) [33]. The other nucleotides are from the flanking sequence. Motifs with an asterisk were identified from phylogenetic analyses. IUPAC nucleotide code: R (A or G); Y (C or T); S (G or C); W (A or T); K (G or T); M (A or C); B (C or G or T); D (A or G or T); H (A or C or T); V (A or C or G); N (any base). [file 1471-2164-13-482-S2.docx]

**Additional file 2. *ANT* gene models in mammals**

|  | **Motif 1** | **Motif 2** | **Motif 3** | **Motif 4** | **Motif 5** | **Motif 6** |
| --- | --- | --- | --- | --- | --- | --- |
| **ANT1** | V$MEF2*  TAAAAATAACYCY | V$GATA*  CWGATAAR | V$NRF1  VNCGCGCABGCGC | (TSS) | ATG | - |
|  | V$GATA  CWGATAAR | V$NRF2.01  acc**GGAA**gns | CAAT | TATAA | (TSS) | ATG |
|  | OXBOX  GGCTCAAAGAGG | V$GATA1.04*  cw**GATA**aca | CAAT | O$VTATA.01  sta**TAAA**wr | (TSS) | ATG |
|  | V$MEF2*  TAAAAATAACYCY | V$GREF*  GGTWCWNNNTGTTCTNR | V$GATA*  CWGATAAR | V$ETSF*  VSMGGAAGYG | (TSS) | **-** |
|  | V$MEF2*  TAAAAATAACYCY | V$GREF*  GGTWCWNNNTGTTCTNR | V$GATA*  CWGATAAR | V$KLFS*  SNGGGTGKGGCN | V$STAT*  TTMYGGGAA | **(**TSS) |
| **ANT2** | GRBOX  CATTGTTATGTT | V$MZF1.01  gn**GGGG**a | V$EGR1.02  ssskgngg**GGGC**gknd | V$SP1.03  g**GGGC**gggg | TATAAA | (TSS) |
|  | GRBOX  CATTGTTATGTT | V$MZF1.01  gn**GGGG**a | V$EGR1.02  ssskgngg**GGGC**gknd | V$SP1.01  sg**GGGC**gggg | O$VTATA.01  sta**TAAA**wr | (TSS) |
|  | V$HOXF*  KTAATTAGCTNA | V$CEBP*  TTGTGMAA | V$MYT1*  AAAGTTTACTT | V$PARF*  ATTRYGTAAC | V$GATA*  CWGATAAR | (TSS) |
| **ANT3** | V$CTCF*  CSCNNSGCCGCK-  -RGGGGNNGSB | V$CHRE*  CACGNGGNV-  -NNCNCGTG | V$RXRF*  RGGTCAKN-  -NAAGTTCA | V$RORA*  WAWNTAG-  - GTCAGKG | ATG | (TSS) |
|  | V$CTCF*  CSCNNSGCCGCK-  -RGGGGNNGSB | V$CHRE*  CACGNGGNV-  -NNCNCGTG | V$RXRF*  RGGTCAKN-  -NAAGTTCA | (TSS) | - | - |
| **ANT4** | V$SMAD3.01*  **GTCT**gg | V$MZF1.02*  dd**GGGG**a | V$HIFF*  HSBCGBACGTGNS | V$HIFF*  HSBCGBACGTGNS | (TSS) | (ATG) |
|  | V$SMAD3.01*  **GTCT**gg | V$MZF1.02*  dd**GGGG**a | V$MAZ.01*  kgs**GAGG**ggag | V$HIFF*  HSBCGBACGTGNS | V$HIFF*  HSBCGBACGTGNS | (TSS) |
|  | V$HMGA.01*  dkcsnnrtn**AAT**Kank | V$ETS2.01*  dac**AGGA**aryvnkt | V$HIFF*  HSBCGBACGTGNS | V$HIFF*  HSBCGBACGTGNS | (TSS) | **-** |
|  | V$SORY*  RAACAAAGRA | V$ETSF*  VSMGGAAGYG | V$HIFF*  HSBCGBACGTGNS | V$HIFF*  HSBCGBACGTGNS | (TSS) | - |

Matrix families (i.e. V$MEF2), matrices (i.e. V$NRF2.01) and IUPAC strings from the four human ANTs gene promoters. For matrix families, an example of IUPAC string is shown. For matrices, only nucleotides with high information content are presented (the matrix exhibits a high conservation at this position). For matrices, nucleotides in bold capital letters denote the core sequence used by MatInspector (defined as the highest, usually four, conserved consecutive positions) [1]. All the IUPAC sequences and the core sequences of matrices (in bold) are searched with 100 % of identity and the flanking sequence with a threshold of 90 %. The other nucleotides are from the flanking sequence. Motifs with an asterisk were identified from phylogenetic analyses. IUPAC nucleotide code: R (A or G); Y (C or T); S (G or C); W (A or T); K (G or T); M (A or C); B (C or G or T); D (A or G or T); H (A or C or T); V (A or C or G); N (any base).

**ANT1:** - V$MEF2, bound by the myocyte-specific enhancer factor, is involved in a mechanism conserved from flies to mammals to fine-tune gene expression in each muscle and probably other tissues [2]; - V$GATA, such as GATA4 element-binding factors, controls cardiomyocyte proliferation by regulating numerous genes involved in the cell cycle [3]. GATA4 also controls a regulatory pathway that regulates PGC-1alpha gene expression in skeletal muscle [4]; - matrices from the V$GREF family are glucocorticoid responsive and related elements [5]; - V$ETSF includes human and murine ETS1 factors [6]; - V$NRF1 is bound by nuclear respiratory factor 1, which is a bZIP transcription factor that acts on nuclear genes encoding mitochondrial proteins through MEF2 (myocyte enhancer factor 2A) [7]; - V$NRF2.01, bound by the nuclear respiratory factor 2 (ETS1 factors), activates the PDGF-α transcription in smooth muscle cells [8]; - V$KLFS Krueppel like transcription factors are critical regulators of cell differentiation including skeletal and smooth muscle development [9]. V$STAT (signal transducer and activator of transcription) is involved in a signalling cascade with a crucial role in regulating myogenesis [10]. The OXBOX motif is a positive transcriptional element of the heart-skeletal muscle ADP/ATP translocator gene [11].

**ANT2:** - V$HOXF, bound by HOX genes 1 to 8, controls normal development and primary cellular processes involved in carcinogenesis [12]; - V$MZF1.01 (myeloid zinc finger protein MZF1) is involved in cell proliferation and its abnormal expression results in cancer development [13]; - V$EGR1.02 (EGR1, early growth response 1) is involved in regulating homeostasis of hematopoietic stem cells (HSC) by coordinating proliferation and migration [14]; - V$SP1.01 and .03 (stimulating proteins) are known to regulate lipogenesis and proliferation in cancer cells [15]; - V$CEBP (ccaat/Enhancer Binding Proteins) family is known to play a role in cell growth and proliferation [16]; V$MYT1 is bound by the myelin transcription factor 1 (Myt1), a zinc finger DNA-binding protein that regulates cell proliferation and differentiation of oligodendrocyte lineage cells [17]; - V$PARF (PAR/bZIP family), including the Drosophila PAR domain protein I gene (Pdp1), regulator of larval growth, mitosis and endoreplication, plays a critical role in coordinating growth and DNA replication [18]; - V$GATA factors control the development of diverse tissues and are involved in mechanisms of carcinogenesis apart from their normal functions [19].

**ANT3:** - V$CTCF (CTCF and BORIS gene family); V$CHRE (carbohydrate response elements); V$RXRF (RXR heterodimer binding sites); V$RORA (v-ERB and RAR-related orphan receptor alpha) needed stringencies too low to lead to conclusive results for the ANT3 gene regulation (Supplementary Table S4).

**ANT4:** - V$SMAD3.01 (bound by the Smad3 transcription factor) determines androgen responsiveness and is involved in testes development [20]. - V$MZF1.02 is recognized by the myeloid zinc finger protein MZF1 [13]; - the V$HMGA.01 matrix is included in the HMGA family of architectural transcription factors [21]; - V$SORY includes SOX/SRY-sex/testis determining and related HMG box factors [22]; - Matrices from the V$ETSF family show an interaction with an androgen receptor [23]; - V$MAZ.01 is recognized by a Myc associated zinc finger protein (MAZ); - V$HIFF: A specific nucleotide string, CACGTGTCAGG, is present in several copies (three in humans) spaced 3 nucleotides apart, with the final string located 33 nt upstream of the transcription start nucleotide (TSS). This final nucleotide sequence is shared by several matrices from the V$HIFF families, including HIF1-alpha/ARNT heterodimers). Such matrices are involved in the glycolytic pathway [24], as suggested during spermatogenesis [25].

[1] K. Quandt, K. Frech, H. Karas, E. Wingender, T. Werner, MatInd and MatInspector: new fast and versatile tools for detection of consensus matches in nucleotide sequence data, Nucleic Acids Res. 23 (1995) 4878-4884.

[2] H. Feng, T. Cheng, J.H. Steer, D.A. Joyce, N.J. Pavlos, C. Leong, J. Kular, J. Liu, X. Feng, M.H. Zheng, J. Xu, Myocyte enhancer factor 2 and microphthalmia-associated transcription factor cooperate with NFATc1 to transactivate the V-ATPase d2 promoter during RANKL-induced osteoclastogenesis, J. Biol. Chem. 284 (2009) 14667-14676.

[3] A. Rojas, S.W. Kong, P. Agarwal, B. Gilliss, W.T. Pu, B.L. Black, GATA4 is a direct transcriptional activator of cyclin D2 and Cdk4 and is required for cardiomyocyte proliferation in anterior heart field-derived myocardium, Mol. Cell. Biol. 28 (2008) 5420-5431.

[4] I. Irrcher, V. Ljubicic, A.F. Kirwan, D.A. Hood, AMP-activated protein kinase-regulated activation of the PGC-1alpha promoter in skeletal muscle cells, PLoS One 3 (2008) e3614.

[5] C.C. Nelson, S.C. Hendy, R.J. Shukin, H. Cheng, N. Bruchovsky, B.F. Koop, P.S. Rennie, Determinants of DNA sequence specificity of the androgen, progesterone, and glucocorticoid receptors: evidence for differential steroid receptor response elements, Mol. Endocrinol. 13 (1999) 2090-2107.

[6] A. Hultgardh-Nilsson, B. Cercek, J.W. Wang, S. Naito, C. Lovdahl, B. Sharifi, J.S. Forrester, J.A. Fagin, Regulated expression of the ets-1 transcription factor in vascular smooth muscle cells in vivo and in vitro, Circ. Res. 78 (1996) 589-595.

[7] B. Ramachandran, G. Yu, T. Gulick, Nuclear respiratory factor 1 controls myocyte enhancer factor 2A transcription to provide a mechanism for coordinate expression of respiratory chain subunits, J. Biol. Chem. 283 (2008) 11935-11946.

[8] M.R. Bonello, Y.V. Bobryshev, L.M. Khachigian, Peroxide-inducible Ets-1 mediates platelet-derived growth factor receptor-alpha gene transcription in vascular smooth muscle cells, Am. J. Pathol .167 (2005) 1149-1159.

[9] S.K. Swamynathan, Kruppel-like factors: three fingers in control, Hum. Genomics 4 (2010) 263-270.

[10] M.K. Trenerry, P.A. Della Gatta, D. Cameron-Smith, JAK/STAT signaling and human in vitro myogenesis, BMC Physiol. 11 (2011) 6.

[11] K. Li, J.A. Hodge, D.C. Wallace, OXBOX, a positive transcriptional element of the heart-skeletal muscle ADP/ATP translocator gene, J. Biol. Chem. 265 (1990) 20585-20588.

[12] C. Cillo, G. Schiavo, M. Cantile, M.P. Bihl, P. Sorrentino, V. Carafa, D.A. M, M. Roncalli, S. Sansano, R. Vecchione, L. Tornillo, L. Mori, G. De Libero, J. Zucman-Rossi, L. Terracciano, The HOX gene network in hepatocellular carcinoma, Int. J. Cancer 129 (2011) 2577-2587.

[13] G. Mudduluru, P. Vajkoczy, H. Allgayer, Myeloid zinc finger 1 induces migration, invasion, and in vivo metastasis through Axl gene expression in solid cancer, Mol. Cancer Res. 8 (2010) 159-169.

[14] J.T. DeLigio, D.A. Zorio, Early growth response 1 (EGR1): a gene with as many names as biological functions, Cancer Biol. Ther. 8 (2009) 1889-1892.

[15] S. Lu, M.C. Archer, Sp1 coordinately regulates de novo lipogenesis and proliferation in cancer cells, Int. J. Cancer 126 (2010) 416-425.

[16] G.D. Lu, C.H. Leung, B. Yan, C.M. Tan, S.Y. Low, M.O. Aung, M. Salto-Tellez, S.G. Lim, S.C. Hooi, C/EBPalpha is up-regulated in a subset of hepatocellular carcinomas and plays a role in cell growth and proliferation, Gastroenterology 139 (2010) 632-643, 643 e631-634.

[17] J.A. Nielsen, J.A. Berndt, L.D. Hudson, R.C. Armstrong, Myelin transcription factor 1 (Myt1) modulates the proliferation and differentiation of oligodendrocyte lineage cells, Mol. Cell. Neurosci. 25 (2004) 111-123.

[18] K.L. Reddy, M.K. Rovani, A. Wohlwill, A. Katzen, R.V. Storti, The Drosophila Par domain protein I gene, Pdp1, is a regulator of larval growth, mitosis and endoreplication, Dev. Biol. 289 (2006) 100-114.

[19] R. Zheng, G.A. Blobel, GATA Transcription Factors and Cancer, Genes Cancer 1 (2010) 1178-1188.

[20] C. Itman, C. Wong, B. Hunyadi, M. Ernst, D.A. Jans, K.L. Loveland, Smad3 dosage determines androgen responsiveness and sets the pace of postnatal testis development, Endocrinology 152 (2011) 2076-2089.

[21] L. Borrmann, B. Seebeck, P. Rogalla, J. Bullerdiek, Human HMGA2 promoter is coregulated by a polymorphic dinucleotide (TC)-repeat, Oncogene 22 (2003) 756-760.

[22] V.R. Harley, M.J. Clarkson, A. Argentaro, The molecular action and regulation of the testis-determining factors, SRY (sex-determining region on the Y chromosome) and SOX9 [SRY-related high-mobility group (HMG) box 9], Endocr. Rev. 24 (2003) 466-487.

[23] C.E. Massie, B. Adryan, N.L. Barbosa-Morais, A.G. Lynch, M.G. Tran, D.E. Neal, I.G. Mills, New androgen receptor genomic targets show an interaction with the ETS1 transcription factor, EMBO Rep. 8 (2007) 871-878.

[24] M. Heikkila, A. Pasanen, K.I. Kivirikko, J. Myllyharju, Roles of the human hypoxia-inducible factor (HIF)-3alpha variants in the hypoxia response, Cell. Mol. Life. Sci. 68 (2011) 3885-3901.

[25] H.H. Marti, D.M. Katschinski, K.F. Wagner, L. Schaffer, B. Stier, R.H. Wenger, Isoform-specific expression of hypoxia-inducible factor-1alpha during the late stages of mouse spermiogenesis, Mol. Endocrinol. 16 (2002) 234-243.
