# Supplementary material for: Computational identification of transcriptionally co-regulated genes, validation with the four ANT isoform genes
Source: BMC Genomics. 2012 Sep 15;13:482. doi: 10.1186/1471-2164-13-482 (PMC3477019; doi:10.1186/1471-2164-13-482)
Supplement: Additional file 3 — Scatter plots of the expression levels of genes with identical promoter models with the ANT promoters in different tissues.ANT1: average expression levels of the genes from Table 2 in the lung, skin and brain tissues versus their expression in muscle. ANT2: average expression levels of the genes from Table 3 in normal brain tissue versus their expression in glioblastoma. ANT3: average expression levels of the genes from Additional file 5 in normal brain tissue versus their expression in glioblastoma. ANT4: average expression levels of the genes from Table 4 in the lung, skin and brain tissues versus their expression in testis. Red diamonds correspond to over-expressed genes with at least a 1.5 fold changes between tissues. [file 1471-2164-13-482-S3.docx]

**Additional file 4. Expression levels of genes that share promoter models with *ANT* genes in different tissues**

***ANT1***

***ANT2***

***ANT4***

Expression levels of genes that share promoter models with ANT promoters in different tissues. Data were obtained from hybridization experiments using Affymetrix genechips as described in material and methods. Y scale in log2 of the average values of the ratios. An average value of the ratios was also calculated for each gene whose expression was assessed by several probe sets.

**ANT1**: data are reported for muscle, lung, skin and brain normal tissues. The ratios of the expression levels of the genes in the muscle versus the average expression in the three other normal tissues (lung, skin and brain) were calculated for each probe sets. **ANT2:** The ratios of the expression levels of the genes in glioblastoma versus their expression levels in normal brain tissue were calculated for each probe sets. **ANT4:** The ratios of the expression levels of the genes in testes versus the average expression in the three other normal tissues (lung, skin and brain) were calculated for each probe sets.
