# Supplementary material for: Computational identification of transcriptionally co-regulated genes, validation with the four ANT isoform genes
Source: BMC Genomics. 2012 Sep 15;13:482. doi: 10.1186/1471-2164-13-482 (PMC3477019; doi:10.1186/1471-2164-13-482)
Supplement: Additional file 4 — Genes co-regulated with the ANT3 gene. The full set of results obtained from the analysis with all constructed models of the ANT3 promoter regions were screened as described in Figure 1 either on the whole chromosome human sequences or the human promoter library (results with an asterisk). The gene in bold is shown overexpressed in microarrays. Gene IDs are with 15 numbers (ex. ENSG00000023228). [file 1471-2164-13-482-S4.docx]

**Additional file 5. Scatter plots of the expression levels of genes with identical promoter models with the *ANT* promoters in different tissues**

**ANT1 ANT2**

**ANT3 ANT4**

**ANT1:** average expression levels of the genes from Table 2 in the lung, skin and brain tissues versus their expression in muscle. **ANT2:** average expression levels of the genes from Table 3 in normal brain tissue versus their expression in glioblastoma. **ANT3:** average expression levels of the genes from Additional Table 3 in normal brain tissue versus their expression in glioblastoma. **ANT4:** average expression levels of the genes from Table 4 in the lung, skin and brain tissues versus their expression in testis. Red diamonds correspond to over-expressed genes with at least a 1.5 fold changes between the tissues.
