# Supplementary material for: Computational identification of transcriptionally co-regulated genes, validation with the four ANT isoform genes
Source: BMC Genomics. 2012 Sep 15;13:482. doi: 10.1186/1471-2164-13-482 (PMC3477019; doi:10.1186/1471-2164-13-482)
Supplement: Additional file 5 — Expression levels of genes that share promoter models with ANT genes in different tissues. Expression levels of genes that share promoter models with ANT promoters in different tissues. Data were obtained from hybridization experiments using Affymetrix genechips as described in material and methods. Y scale in log2 of the average values of the ratios. An average value of the ratios was also calculated for each gene whose expression was assessed by several probe sets. [file 1471-2164-13-482-S5.docx]

**Additional file 3. Genes coregulated with the ANT3 gene**

| **Gene,**  **ID** | **Encoded protein**  **(EnsEMBL)** | **Protein function**  **(Uniprot)** |
| --- | --- | --- |
| APC2*  115266 | Adenomatous polyposis coli protein 2 | Promotes rapid degradation of CTNNB1 and function as a tumor suppressor |
| CSAG1  198930 | Chondrosarcoma-associated gene 1 protein | Unknown |
| EFNA2*  99617 | Ephrin-A2 | Unknown, binds to the receptor tyrosine kinases EPHA3, 4 and 5 |
| **IFT88***  32742 | Intraflagellar transport protein 88 homolog | Involved in primary cilium biogenesis |
| LIPC*  166035 | Hepatic triacylglycerol lipase | Hepatic lipase hydrolyses phospholipids, glycerides, and acyl-CoA thioesters |
| MYEOV  172927 | Myeloma-overexpressed gene protein | Unknown, overexpressed in tumor cells lines with a t(11;14)(q13;q32) translocation |
| NR1D2*  174738 | Nuclear receptor subfamily1 groupD member2 | Acts as a competitive repressor of ROR alpha function |
| RNF166*  158717 | RING finger protein 166 | Unknown |
| STMN3*  197457 | Stathmin-3 | Unknown, neuron specific |
| TTI1*  101407 | TEL2-interacting protein 1 homolog | Promotes assembly, stabilizes the activity of mTORC1 and mTORC2 complexes |

The full set of results obtained from the analysis with all constructed models of the *ANT3* promoter regions were screened as described in Figure 1 either on the whole chromosome human sequences or the human promoter library (results with an asterisk). The gene in bold is shown overexpressed in microarrays. Gene IDs are with 15 numbers (ex. ENSG00000023228).
